# Diagnosis of Obstructive Sleep Apnea in Patients with Allergic and Non-Allergic Rhinitis

**DOI:** 10.3390/medicina56090454

**Published:** 2020-09-08

**Authors:** Annalisa Pace, Giannicola Iannella, Valeria Rossetti, Irene Claudia Visconti, Giampiero Gulotta, Carlo Cavaliere, Andrea De Vito, Antonino Maniaci, Salvatore Cocuzza, Giuseppe Magliulo, Andrea Ciofalo

**Affiliations:** 1 Organi di Senso Department, Sapienza University of Rome, Via Gregorio VII n.80, 00165 Rome, Italy; annalisapace90@gmail.com (A.P.); giannicolaiannella@hotmail.it (G.I.); valeria.rossetti92@gmail.com (V.R.); ireneclaudia.visconti@gmail.com (I.C.V.); giampierogulotta@gmail.com (G.G.); carlo.cavaliere@uniroma1.it (C.C.); andrea.ciofalo@uniroma1.it (A.C.); 2 Otorinolaringoatria Department, Santa Maria delle Croci Hospital of Ravenna, Viale Vincenzo Randi, 5, 48121 Ravenna, Italy; dr.andrea.devito@gmail.com; 3 Otorinolaringoiatria Department, University of Catania, Piazza Università, 2, 95131 Catania, Italy; tnmaniaci29@gmail.com (A.M.); scocuzza@unict.it (S.C.)

**Keywords:** OSA, NARES, AR, polysomnography, AHI, nasal obstruction

## Abstract

*Background and objectives*: Rhinitis could be considered a risk factor for obstructive sleep apnea (OSA). Studies were conducted to evaluate the relation between OSA and Allergic rhinitis (AR). Non-allergic rhinitis with eosinophilia syndrome (NARES) is a condition with a symptomatology apparently similar to AR. The aim of this study was to evaluate the different presence of OSA in patients suffering from NARES and AR. *Materials and Methods*: Sixty patients were enrolled and subdivided into NARES, AR and control groups. NARES and AR diagnosis were performed using ARIA (Allergic Rhinitis and its Impact on Asthma) protocol. All patients were screened for OSA with home sleep apnea testing (HSAT) exam analyzing AHI (Apnea Hypopnea Index) values. *Results*: Results showed that 60% of patients affected by NARES presented OSA. On the contrary, altered AHI was found only in 35% of patients affected by AR and in 10% of patients belonging to the control group. *Conclusions*: In conclusion, data showed that there was an increased risk of OSA in NARES patients respect to AR patients and healthy patients.

## 1. Introduction

Allergic rhinitis (AR) represents a global health problem, affecting 10% to 20% of the world population and is defined as an inflammatory process of the nasal mucosa, aroused by environmental allergens. This condition is IgE-mediated and presents inflammatory cells in the mucosa and submucosa [1].

On the other hand, Non-allergic rhinitis with eosinophilia syndrome (NARES) is an infrequent condition defined by a chronic inflammation of nasal mucosa with a prevalence of eosinophilic cells. NARES affects from 13% to 33% of patients with non-allergic rhinitis and usually in vivo and in vitro tests for allergic rhinitis (AR) diagnosis are negative for this specific condition [2,3,4,5].

OSA disease is a common disorder characterized by the of repeated upper airway collapse during sleep, leading to oxygen desaturation and disrupted sleep. It involves about 1 billion people with prevalence exceeding 50% in some countries [6,7,8,9,10,11,12,13].

In the current literature, little data are available regarding the relationship between the different role of AR and NARES on OSA disease [14,15].

Nasal congestion generally increases nasal resistance and reduces nasal airflow during the night, thus producing a mounting negative pressure within the inferior airways (Starling’s resistor model). Along with this, a higher total air flow resistance due to unstable oral breathing, decreased activation of the nasal receptors with consequent inhibition of the muscular tone and baseline breathing frequency (naso-ventilatory reflexes), as well as a reduced level of pulmonary nitric oxide occur, and may result in a respiratory disorder. Finally, posterior collapse of the tongue due to oral breathing could be the cause of secondary palatal obstruction [16,17,18,19,20].

Therefore, as suggested by different authors, patients with allergic rhinitis (AR) and non-allergic rhinitis (non-AR) present an increased risk of snoring and sleep-disordered breathing, as well as longer and more frequent hypopnea/apnea events [9,20].

Nevertheless, the different etiopathogenetic inflammatory mechanisms of NARES and AR could affect in different ways the risk of OSA. The role of AR as a risk factor for OSA is a source of debate.

Patients affected by OSA with associated AR show significant daytime sleepiness and higher ESS (Epworth Sleepiness Scale) score when compared with patients not suffering from rhinitis [18,19,20] and some studies have investigated the presence of AR in OSA patients showing a prevalence of 25% in adult patients [21,22,23,24]. On the contrary, other authors investigated the effect of AR on sleep quality and the severity of OSA, showed that AR did not modify polysomnographic parameters in comparison to other patients without any nasal inflammation [25].

There are few studies regarding the relationship between NARES and OSA that suggested how NARES might stimulate alveolar hypoventilation, resulting in an elevated hypopnea index [26].

The aim of this study was to determine the different incidence of OSA in patients affected by AR and NARES. Severity and sleep disorder characteristics were compared between the two groups of patients, those with rhinitis and those without.

## 2. Materials and Methods

This prospective study was completed at the Rhinology Unit of Organi di Senso Department, “Sapienza” University of Rome between October 2018 and November 2019.

All patients evaluated in this unit due to nasal and rhinitis symptoms were initially considered as possible candidates for the study. All subjects underwent an Otorhinolaryngological physical examination and a fiber-optic endoscopic evaluation of the upper airways [27,28,29,30].

Asthma, chronic obstructive pulmonary disease, corticosteroid and antihistaminic ongoing therapies were considered as study exclusion criteria. Moreover, patients with unclear characteristics such as local allergic rhinitis in NARES and patients using a CPAP therapy were excluded from the study.

Patients with a diagnosis of NARES and AR were subsequently considered eligible for this study. Diagnosis of NARES and AR were performed in these patients using the protocol provided by the ARIA (Allergic Rhinitis and its Impact on Asthma) classification that involves the execution of skin prick test, RAST and nasal cytological [11]. Besides, a control group of patients without rhinitis was realized with subjects negative to a skin prick test, RAST, nasal cytological and SNPT tests.

Sixty subjects were finally enrolled in study in according to above study design and exclusion/inclusion criteria.

The patients were sub-classified according to their rhinology diagnosis into three homogeneous groups of 20 patients: (1) the NARES group, (2) the AR group, and (3) control group (healthy patients without rhinitis).

The characteristics of enrolled patients (i.e., age, gender, BMI, history of gastro-esophageal reflux, previous obesity, smoking habits) were collected and analyzed. Nasal and non-nasal symptom severity (NNNSS) was investigated.

All patients of the three groups were tested for OSA with specific questionnaire to evaluate the OSA risk and a home sleep apnea testing (HSAT) study.

**Figure 1:**
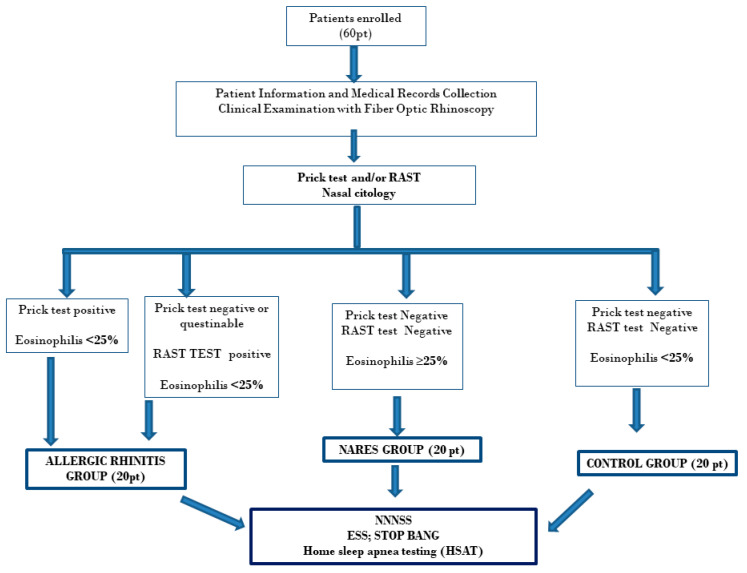
Flow Chart of the material and methods.

A flow chart of study design has been summarized in Figure 1.

All patients were asked to sign an informed consent in order to participate in the study. This study was completed in accordance with the Helsinki Declaration, was approved by the Local Ethics Committee of “Sapienza” University and was conducted according to good clinical practice.

The test used for the selection and evaluation of patients has been reported below.

### 2.1. Allergic Rhinitis Diagnosis

A panel of allergens consisting of Dermathophagoides pteronissinus, Parietaria officinalis, Mix grasses, Alternaria tenuis, Aspergillus fumigatus, Olea, Cupressus, Composite Mix, Dog, Cat, Hazel (Anallergo, Scarperia and San Piero-Florence, Italy) were used to initially evaluate patients for allergic rhinitis. As suggested by the ARIA classification, patients with questionable or negative results underwent RAST and specific nasal provocation test (SNPT) for Dermathophagoides pteronissinus, Parietaria officinalis and Alternaria tenuis [11].

### 2.2. NARES Diagnosis

Nasal cytology was used to obtain the NARES diagnosis [11]. Nasal smears were collected by scraping the medial surface of the inferior turbinate. The material collected was spread on a glass slide and fixed with cytologic fixative. The material was stained with May–Grunwald–Giemsa stain (C. Erba, Milan, Italy), mounted, cover slipped and observed under optical microscopy BM100FL (Seben, Berlin, Germany). The eosinophil count was performed on 10 immersion microscope fields (×1000) and was expressed as a percentage of the total cell count.

NARES was diagnosed when there were ≥25% eosinophils in patients with negative in vivo and in vitro allergological tests [31,32].

### 2.3. Non-Nasal Symptom (NNSS) Severity Investigation

NNNSS evaluation was performed by asking patients to assess the intensity (acuity) of their individual symptoms using a visual analogue scale. A score was assigned varying from 1 (absent or occasional episodes) to 7 (maximum acuity) to each of the five nasal symptoms (sneezing, runny nose, congestion, itchy nose, post nasal drip) and to non-nasal symptoms (chronic cough, throat symptoms, headache), in accordance with the procedures of the American Academy of Allergy Asthma and Immunology and the Joint Council of Allergy, Asthma and Immunology [33,34,35].

### 2.4. STOP BANG and ESS

The subjects of all three group were asked to answer the STOP-BANG (S = snoring, T = tiredness, O = observed stop breathing, P = pressure, B = BMI, A = age, N = neck, G = gender) questionnaire. This is a screening method used for identifying individuals at risk for OSA [36]. STOP-BANG consisted of 8 yes/no questions based upon snoring, tiredness/sleepiness, apneas, high blood pressure, BMI > 35 kg/m^2^, age > 50 years, neck circumference >40 cm and male gender. This is a validated test and is a risk index for OSA. A score ≥3 was shown to have a high sensitivity for detecting OSA. A score of 5–8 identified patients with a high probability of developing moderate/severe OSA [37].

The ESS was used in all groups for assessing excessive daytime sleepiness. The ESS is a self-administered questionnaire for assessing individual changes in sleepiness in the afore-mentioned eight activities of daily living. For each item, changes in sleepiness were recorded on a scale of 0–3 (0 = none, 1 = slight, 2 =moderate or 3 = high change). Thus, the overall ESS score ranged from 0 to 24. A total score ≥ 10 is consistent with excessive daytime sleepiness [38].

### 2.5. Home Sleep Apnea Testing (HSAT)

All participants of the study underwent one-night home sleep apnea testing, type III portable monitor (HSAT; Embletta MPR) as defined in the AASM rules.

The following parameters were recorded during the sleep study: respiratory movement and airflow, heart rate, arterial oxygen saturation, patient’s position and sleep time.

Obstructive apnea is associated with continued or increased inspiratory effort throughout the entire period of absent airflow.

Obstructive apnea is defined by absence of airflow for at least 10 s with a continued or increased inspiratory effort throughout event.

Hypopnea is characterized by all these criteria: peak signal excursion drops by >30% of pre event baseline; the duration of the >30% drop in signal is >10 s; there is a >3% oxygen desaturation from pre event baseline.

In accordance with the AASM Manual for the Scoring of Sleep and Associated Events, OSA severity was classified on the basis of the apnea + hypopnea index (AHI) [39,40].

The grade of OSA was classified as normal (AHI < 5/h), mild (AHI ≥ 5 and < 15 plus typical symptomatology), moderate (AHI ≥ 15 and < 30) or severe (AHI ≥ 30) [41].

The reports were evaluated by the same sleep medicine specialist.

### 2.6. Statistical Analysis

Descriptive data were expressed as counts (percentage) for groups of patients. Comparison between the groups was performed using Mood’s Median Test for continuous variables and Chi square (X2) test for categorical variables. Statistical probability was verified with Chi square (X2) or Fisher exact test, depending on the number of observations. ANOVA Test was performed. A *p* value < 0.05 was considered significant. Statistical analysis was performed using the Number Cruncher Statistical System 10 program (NCSS, Kaysville, UT, USA).

## 3. Results

Demographic and clinical data are reported in Table 1. No differences between the NARES, AR group and control group were identified when considering the variables of age, BMI, pre-existing gastro-esophageal reflux disorder and smoking habits (Table 1).

### 3.1. Nasal Cytology

Nasal cytology testing showed that all twenty patients enrolled in NARES group presented pathological percentage of eosinophils that have an average value of 42.5%. In patients belonging to the AR group, the same test showed inferior values (5.6%) (Table 2). The different eosinophilic nasal concentration between the two groups was statistically significant *p* < 0.001.

The group of patients affected by AR resulted positive to skin-testing for *Dermatophagoides pteronyssinus* (12 patients) or for *Parietaria officinalis* (8 patients).

Nasal cytology testing conducted in control patients did not presented eosinophils. Furthermore, skin testing results were negative.

### 3.2. NNNSS

**Figure 2:**
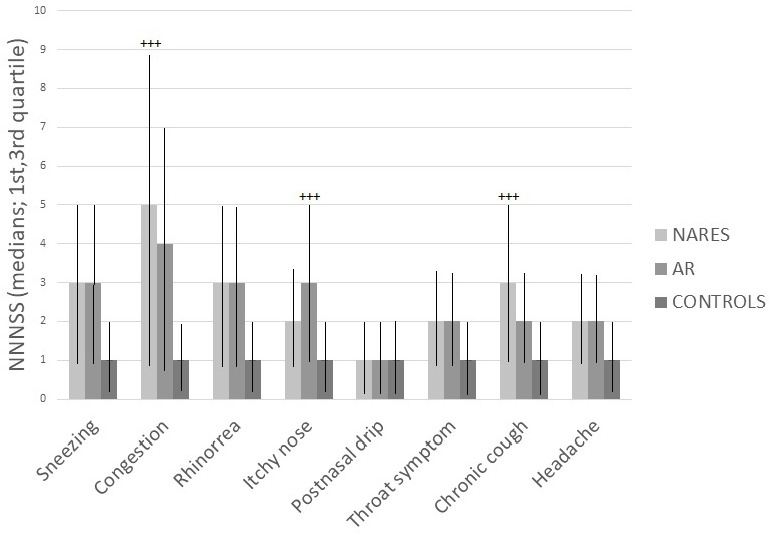
Nasal and non-nasal symptom severity (NNNSS) values for the three groups. These values are expressed as a median with first and third quartile. (+++: more statistically significant values).

Figure 2 reports the NNNSS values for the three groups. These values are expressed as a median with first and third quartile. Nasal congestion and chronic cough were found to be prevalent symptoms in the group of NARES, whereas nasal itch prevailed in the group with AR. The comparison between NARES vs. AR scores showed that median values for the various symptoms were the following (Table 2): 5 vs. 4 for nasal congestion, *p* = 0.001; 3 vs. 2 for chronic cough (*p* = 0.001) and 2 vs. 3 (*p* = 0.001) for nasal itch. The comparison with the control group was statistically significant for all symptoms (*p* < 0.0005) with the exclusion of postnasal drip.

### 3.3. STOP-BANG and ESS

Using the STOP-BANG questionnaire, 80% of patients with NARES showed a score of 5–7 (indicating the possibility of mild/severe OSA), whereas 60% of patients with AR showed a score of 3–4 (indicating the possibility of mild grade OSA). In total, 11% of the patients in the control group presented a score of 1 (0–1).

ESS average value expressing the risk of OSA, as measured by the STOP-BANG, was higher in the NARES group than in the AR one (6 vs. 4, *p* < 0.0025. Table 2) and in the control group (6 vs. 1, *p* < 0.0005).

The NARES group showed a higher ESS score than the AR group (14.5 vs. 10.5, *p* < 0.005) and the control group (14.5 vs. 4, *p* < 0.0005) (Table 2).

### 3.4. Home Sleep Apnea Testing (HSAT)

OSA was diagnosed in 60% of patients with NARES. Of these patients, 42% presented mild OSA, 25% had moderate OSA and 33% severe OSA.

In the AR group, 35% of patients evidenced AHI > 5. Of these, 57% had mild OSA, 14% moderate OSA and 29% severe OSA.

In the control group, 10% of patients were affected by OSA. In total, 50% of these patients showed mild OSA and 50% presented moderate OSA; none presented severe OSA.

Results of Chi square (X2) test disclosed a statistical difference between the prevalence of OSA only in NARES group vs. Control One (*p* < 0.005). On the contrary, there was no statistical difference for OSA between NARES group and AR one and between AR group and Control one (*p* > 0.005).

Comparison of prevalence between subclasses of severity of OSA, in the different groups, showed that there was no statistical difference (*p* > 0.005) (Table 3).

The AHI average value of patients affected by OSA in NARES groups was 19.1. In the AR group the AHI average value was 16.3, while it was 15.5 in control one. There was no statistical difference comparing AHI of three groups (*p* > 0.005).

ANOVA TEST conducted showed F ratio = 9.55 (F crit 3,15) and a statistical significative different between groups (*p* < 0.0003).

## 4. Discussion

In the literature, there are many articles that evaluate AR as a potential risk for OSA, but few studies have been conducted regarding the relationship between OSA and NAR and, in particular, about OSA and NARES [41,42,43,44,45].

The results of this study showed that there was a statistical difference between the prevalence of OSA in NARES group vs. the control one.

On the contrary, there was no statistical difference for OSA between the NARES group and the AR one and between the AR group and the control one.

Moreover, 60% of patients affected by NARES presented OSA vs. 35% of patients affected by AR and 10% of control patients. These results showed that the incidence of OSA in patients with rhinitis, in particular, patients affected by NARES, is higher than in general population. This is in line with the data reported in literature where it is described that AR and NAR are associated with different percentages and grades of OSA.

Cao et al. in their meta-analysis, showed that in adults the incidence of AR in OSA patients was 35.2 [39].

Magliulo et al. in accordance with the ARIA protocol, showed that AR and non-AR conditions were presented in 18% and 26% of OSA patients, respectively. In patients with a diagnosis of AR or NAR, mean AHI did not differ from those not diagnosed with rhinitis (*p* > 0.05 in each case). In the sub-classification of non-AR (NAR), NARES emerged in 4% of the analyzed patients [14].

Similar data were expressed by Zheng et al. They enrolled 240 patients affected by OSA; of these, 134 received a diagnosis of rhinitis: 65 had AR and 69 had NAR. Moreover, 7.5% of patients with AR and 5.4% with NAR demonstrated mild OSA severity, whereas 13.3% of patients with AR and 12.5% with NAR demonstrated high OSA severity [41]. OSA was mainly mild to moderate in AR, with no severe disease, whereas mild sleep apnea was non significantly prevalent in patients with NAR. Moreover, NAR showed a high correlation not only with OSA but also with apneas. Kalpaklioglu, in 2009, evaluated the risk of OSA in patients affected by rhinitis. In the cohort study, 36% of patients affected by AR presented OSA, whereas 86% of patients affected by NAR group presented OSA [27].

On the contrary, the results of our study showed that a higher grade of OSA (moderate to severe) was more common in the NARES group that in the AR and control ones.

This is in line with the data reported in the available literature and suggests that chronic eosinophilic inflammation could support OSA, [26,46] in accordance with the data described by Kramer et al. in their studies. During the first study, conducted in 2001, they tested the relation between OSA symptoms and AR. They enrolled 119 patients who underwent polysomnographic exam and testing for AR diagnosis (in vivo and in vitro). Results were interesting: no significant rates of AR associated with OSA were found. No significant differences in sleeping parameters were observed between allergic and non-allergic patients: 16% of the non-allergic patients had an AHI >10, compared with only 50% of allergic patients. However, there was a small group of patients affected by NARES (3.4% of total population) that mainly presented OSA (75% of patients affected by NARES) [44].

According to this data, in 2004, Kramer studied patients suffering from NARES, revealing significantly altered polysomnographic parameters compared with control patients [26]. In fact, subjects that suffered from NARES presented mainly severe OSA (7/10 patients), while patients without any nasal inflammation suffered from moderate or mild OSA (13/16 patients).

As suggested in the introduction, it is important to remember that AR and NARES have a different pathogenesis. According to this, the differences in OSA incidence observed in our results could be related with the possibility that the only typical symptomatology caused by AR and NARES (nasal itching, sneezing, rhinorrhea and nasal congestion/obstruction) is not sufficient to cause OSA [41].

In the study of 2004, Kramer also demonstrated that polysomnographic exams show a prevalence of hypopnea with respect to apnea; moreover, both groups revealed no significant differences in nasal flow measured by standard rhinomanometry.

The mechanism responsible for the increased presence of OSA in NARES patients compared to AR and healthy subjects is unknown. To explain the latter, Kramer considered neuronal reflex branches (nasal-pulmonary reflex) as a possible cause of alveolar hypoventilation and consequent increase in hypopnea index [26].

The elevated number of patients with AHI > 5 identified in patients with NARES, could also be correlated with a marked bronchial hyper-reactivity and the direct activity of the major basic protein (MBP) on the nasal mucosa or progression to the lower airway of the eosinophils located on the cholinergic innervations [46]. This mechanism could determine an increase in the activity of the neuronal naso-pulmonary reflex with secondary alveolar hypoventilation. The effect of the MBP on the substance P immune reactive nerves seems to facilitate the action of this neurotransmitter in producing vasodilatation, secretion of mucous and plasma exudation as well as an increase in the rapid latency of the ocular roving movements [47,48]. Moreover, neurogenic inflammation seems to contribute to an increased nasal congestion as we highlighted in the NARES group. In addition to toxic basic proteins, the chronic eosinophilic inflammation, more notable and consistent in the NARES group, produces significantly elevated levels of interferon gamma, tumor necrosis factor alfa, interleukin–1beta, interleukin-4 and interleukin–17. All the latter have pro inflammatory activity with an increase in nasal and bronchial hyper-reactivity [49,50]. Some present a sleep-inducing effect, acting within the central nervous system. Others, positively correlate with the degree of local eosinophilia, increasing latency while reducing the frequency of rapid eye movement during sleep in association with decreased latency of sleep initiation and reduction of the diaphragmatic contractility [49,50,51,52,53]. Some limitations are present in this study. The highly selective inclusion criteria, in fact, did not allow us to have a greater number of patients. Moreover, it was decided to exclude diseases such as nasal polyposis and asthma. The sample size is small because of all the co-factors examined. Despite the ANOVA test results showing that there was a statistical difference between the three groups for AHI, the small sample size remained the most significative study limitation. Further studies with larger samples of patients suffering from AR and NARES are under way to confirm these results.

## 5. Conclusions

Nasal congestion, chronic eosinophilic inflammation diffused via circulating cytokines and humoral mediators or via heightened nervous reflexes could increase the risk of OSA. According with this study results, it is possible to hypothesize that NARES, more than AR, OSA could be related with OSA disease. Further studies with larger samples of patients suffering from AR and NARES are under way to confirm these results.

## Figures and Tables

**Table 1:** Demographic and Clinical Data.

Characteristics	Nares 20 pts	AR 20 pts	Controls 20 pts	*p*	*p*′	*p*″
Male %	60 (12/20 pz)	50 (10/20 pz)	50 (10/20 pz)	0.02	0.15	0.25
Female %	40 (8/20 pz)	50 (10/20 pz)	50 (10/20 pz)	0.21	0.02	0.26
Age, mean ± SD, y	47.9 ± 11.3	45.8 ± 8.7	46.6 ± 7.8	0.4	0.35	0.35
Height, mean ± SD, cm	174.8 ± 3.2	173.3 ± 1.9	171 ± 2.25	0.25	0.69	0.75
Weight, mean ± SD, Kg	75.3 ± 4.3	72.5 ± 2.5	69.6 ± 3.8	0.62	0.42	0.9
Gastroesophageal reflux disease, %	15 (3/20 pz)	10 (2/20 pz)	10 (2/20 pz)	0.9	0.38	0.04
Smoker, %	20 (4/20 pz)	25 (5/20 pz)	20 (4/20 pz)	0.14	0.14	0.036
BMI, mean ± SD, Kg/m^2^	23.8 ± 3	23.72 ± 2.1	23.6 ± 1.9	0.23	0.78	0.87
Neck circumference, mean ± SD, cm	40 ± 3	39 ± 2	38 ± 1.5	0.45	0.45	0.39

*p* = NARES vs. AR, *p*’ = NARES vs. Controls, *p*” = AR vs. Controls. (chi square test). Pts = patients; BMI = Body Mass Index NARES = Non-Allergic Rhinitis with Eosinophilia Syndrome. AR = Persistent Allergic Rhinitis.

**Table 2:** Scores (median, 1st and 3rd quartile) of Nasal Eosinophilia, NNNSS, Epworth sleepiness scale (ESS).

	NARES (n = 20)	AR (n = 20)	Controls (n = 20)	*p*	*p*′	*p*″
**Eosinophils (%)**	42.5 (35–48.5)	5.6 (2.5–7.75)	0	0	<0.005	<0.005
**NNNSS**						
Sneezing	3 (1–4)	3 (3–3)	1 (1–1)	0.020	0.0002	0.000005
Congestion	5 (5–6)	4 (3–4)	1 (1–1)	0.001	0.0035	0.00009
Rhinorrhea	3 (3–4)	3 (2–4)	1 (1–1)	0.025	0.0005	0.00066
Itchy nose	2 (1–2.25)	3 (2–3.75)	1 (1–1)	0.001	0.0004	0.00114
Postnasal drip	1 (1–1.25)	1 (1–2)	1 (1–1)	0.01	0.021	0.038
Throat symptom	2 (1.75–2)	2 (1–2)	1 (1–1)	0.026	0.0015	0.00078
Chronic cough	3 (2–4)	2 (2–2)	1 (1–1)	0.001	0.0006	0.0005
Headache	2 (1–3)	2 (1–2)	1 (1–1)	0.07	0.0004	0.0003
**ESS**	14.5 (12.25–18.25)	10.5 (7.5–13.5)	4 (1–5)	0.0006	0.002	0.0026
**STOP-BANG**	6 (5–7)	4 (3.75–5.25)	1 (0–1)	0.004	0.0004	0.041

*p* = NARES vs. AR, *p*′ = NARES vs. Controls, *p*″ = AR vs. Controls. (chi square test). NARES = Non-Allergic Rhinitis with Eosinophilia Syndrome. AR = Persistent Allergic Rhinitis.

**Table 3:** Number and percentage of obstructive sleep apnea patients in the different groups studied.

	NARES		AR		CONTROL		*p*	*p*′	*p*″
	n°	%	n°	%	n°	%			
**NO-OSA**	8/20	40	13	60	18	90		0.0004	0.05
**OSA**	**12/20**	**60**	**7/20**	**35**	**2/20**	**10**	0.05		
*MILD OSA*	5/12	42	4/7	57	1/2	50	0.6	1	1
*MODERATE OSA*	3/12	25	1/7	14	1/2	50	1	0.4	0.5
*SEVERE OSA*	4/12	33	2/7	29	/	/	1	1	1

*p* = NARES vs. AR, *p*’ = NARES vs. Controls, *p*” = AR vs. Controls (chi square test).
